# Gender Diversity in Orthopedics—New Insights and Ongoing Concerns

**DOI:** 10.1007/s00268-023-06963-0

**Published:** 2023-03-24

**Authors:** Megan O’Connor, Marí Thiart

**Affiliations:** 1 Orthopaedics, Harry Gwala Regional Hospital, University of KwaZulu-Natal, Nelson R. Mandela School of Medicine, Durban, South Africa; 2 Orthopaedics, Tygerberg Hospital, Stellenbosch University, Cape Town, South Africa

With underrepresented minority groups and women’s rights movements receiving increasing exposure in popular culture, and traditionally male-dominated sectors such as orthopedic surgery steeped in diversity discourse, one can quickly be desensitized to ongoing concerns. Therefore, periodically revisiting the importance of diversity and reevaluating the reasons for context-specific slow progress, as was the intent of this investigation, is necessary to guide future efforts at change.

Diversity, understood herein to mean differences in gender, as well as cultural, social or ethnic backgrounds of individuals within the workplace, has been associated with improved innovation, creativity and economic growth in business [1, 2]. This effect is attributed to the rich perspectives and insights diversity offers [1]. A diverse group of physicians is better equipped to understand and effectively relate to their patients. Research has found that a diverse health sector enhances patient satisfaction and improves patient access to care [2]. Despite this, female orthopedic surgeons and residents continue to represent a minority, highlighting the importance of determining barriers to enhancing gender diversity in this context [1, 2]. Underrepresentation may be related to a lack of inclusivity. Inclusion is distinct from diversity (the different physical, social, and ethnic characteristics of individuals), and refers to the experience within a diverse group that everyone has value, is treated equitably and ‘belongs.’ Social science investigation has determined that 30% representation is the critical point at which inclusivity can be appreciated [1]. Why this crucial percentage for gender diversity has not been obtained in the Israeli orthopedic residency context was the subject of this investigation.

Utilizing an online questionnaire, the authors surveyed female residents to determine if there were differences in sociodemographic characteristics, exposures to orthopedics, or perceptions of orthopedic residency, between women who chose orthopedic residency training as compared to other specialties [3]. Three of the reported findings were striking because they were either contrasting, corroborative, or ongoing concerns voiced in previous publications.

In stark contrast to previous work on pregnancy and motherhood in orthopedic residents, only 8% of women in this study had *no* children (both orthopedic and non-orthopedic residents). In the unlikely event that all 8% of these women fell into the orthopedic residency group, this would constitute 24% of Israeli female orthopedic residents without children. While not a direct comparison to the figure mentioned above, a 2022 publication investigating pregnancy in orthopedic residents in the US determined that 42% of female residents wished to have a child during residency. Only 22% of residents had children during residency, and 69% of residents deliberately delayed childbearing due to the demands of their residency program [4]. From these figures, one could infer that considerably more than 24% of female orthopedic residents in the US do not have children. Fear of not being able to balance maternal and clinical duties has been reported as a barrier to pursuing a career in orthopedics for US female residents and could partially account for underrepresentation rates there [4]. In the Israeli cohort representation is also low. However, motherhood concerns during residency, while not explicitly explored, appear to play less of a role. Social support structures and other reasons why this may be the case should be explored in future investigation, to provide insight into how this obstacle may be minimized for residents who deem it a barrier.

Secondly, in corroboration with previous investigation of the orthopedic training pipeline, the authors found that more of the female *orthopedic* residents were exposed to orthopedics during medical school than residents in other fields [5]. Mason et al. conducted a summer school training program to expose medical students to orthopedics [5]. Women who participated in the program had increased odds of applying for an orthopedic residency position [5]. The global difference in the representation of women in medical schools, compared to that in orthopedic residencies, suggests that continued efforts need to be focused on female medical students’ exposure to orthopedics, to retain women with an initial interest in the field [1, 2]. Numerous successful initiatives have been implemented based on this premise, including the Perry initiative and Nth Dimensions, the designs of which are easily translatable to other contexts [2]. Van Heest explains further that clinically rotating through orthopedics and appropriate mentorship are of greater import to female medical students than their male counterparts and should also be considered in developing appropriate programs [1].

The concerning and almost uniform finding in studies investigating the gender disparity in orthopedics is the high prevalence of gender discrimination. From subtle micro-aggressions experienced by 74% of female orthopedic surgeons in one study, to overt and explicit sexual harassment [2, 6]. A US-based study found that 81% of female orthopedic surgeons had experienced either bullying, discrimination, sexual harassment or harassment [1]. In the Israeli study, female orthopedic residents perceived discrimination more than twice as often as other residents. Gender discrimination and implicit bias are deeply rooted in culture, and progress here will take time. Tangible changes in discrimination will likely only be appreciated once the 30% critical mass of inclusivity is reached.

What is evident from this article is that some effective strategies developed to increase female representation in orthopedics may be of benefit to all contexts. There are however still new insights to be gained from differing settings. The pervasive implicit biases resulting in gender discrimination will take longer to transform as diversity becomes encultured.
